# The online learning experience and reported headaches associated with screen exposure time among Saudi health sciences students during the COVID-19 pandemic

**DOI:** 10.1186/s12909-022-03235-8

**Published:** 2022-04-01

**Authors:** Ebtsam Aly Abou Hashish, Nada Yasser Baatiah, Alia Hamdi Bashaweeh, Abdullah Mohammad Kattan

**Affiliations:** 1 Present Address: College of Nursing – Jeddah, King Saud bin Abdul-Aziz University for Health Sciences, King Abdulaziz Medical City, National Guard Health Affairs, Jeddah, Saudi Arabia; 2 Faculty of Nursing, Alexandria University, Alexandria, Egypt; 3 College of Applied Medical Sciences, King Saud Bin Abdul-Aziz University for Health Sciences, Jeddah, Saudi Arabia; 4 College of Medicine, King Saud Bin Abdul-Aziz University for Health Sciences, Jeddah, Saudi Arabia

**Keywords:** Headache, Online learning, Screen exposure time, University students, COVID-19 pandemic

## Abstract

**Background:**

The COVID-19 pandemic has become a global health issue and has significantly impacted university education. As a result, learning methods have been shifted to be delivered through online learning. Online learning has increased reliance on computer screens, which can cause visual discomfort and may cause or exacerbate headaches due to prolonged screen exposure. However, time spent using electronic devices has not yet been examined in relation to the online learning experience.

**Purpose:**

This study assessed the online learning experiences and reported headaches associated with screen exposure time among health sciences university students.

**Methods:**

A cross-sectional study was conducted among a convenience sample of 353 students at Saudi University. Online learning experiences, screen time exposure, and reported headache questionnaires were used to collect the data.

**Results:**

Students were moderately satisfied with the online learning experience. Nevertheless, they faced many challenges with online learning that affected their communication efficacy, and they preferred that blended learning be continued. In addition, this study found a high prevalence of headache (65.72%) and a high screen exposure time among the studied students (52.69%). Increased screen time exposure is linked with increased headache and migraine reporting among students (*p* < 0.05).

**Conclusion:**

Headache is a common health issue among health professional students, and it can harm their academic performance and quality of life, especially related to online learning. Greater awareness of headaches, stress reduction and prevention programs, and ergonomic practices to deal with headaches are essential. Blended learning approaches can improve student learning and performance in health science courses.

**Supplementary Information:**

The online version contains supplementary material available at 10.1186/s12909-022-03235-8.

## Introduction

The COVID-19 pandemic has recently become a global health issue and has significantly impacted education. As a result, learning methods were shifted to online learning halfway through the second semester of 2019/2020 [1]. Because of the COVID-19 pandemic, students and teachers in higher education institutions have been subjected to unprecedented changes. In less than a month, online learning replaced traditional face-to-face learning to ensure educational continuity [2, 3].

*Online learning* is defined as an interactive learning experience and education delivered over the internet via electronic devices [4]. To obtain this learning experience, students must use particular online technologies such as Cloud Meetings, Blackboard, and Microsoft Teams. Online learning has recently become an essential part of education worldwide, with many colleges and schools offering online courses to make education more accessible to large populations and provide a less expensive option for people who cannot afford to travel and live abroad [1, 5]. Universities were pushed by the abrupt impact of the COVID-19 pandemic to provide students online educational environments that were both immediately applicable and supportive of quality learning. As a result, a wide range of synchronous and asynchronous online educational environments have been adopted [6].

### Theoretical framework

The two main types of online learning that are usually compared are asynchronous and synchronous online learning. *Synchronous* online learning enables students to use the internet and interact with class materials simultaneously with their classmates. This delivery method allows students to learn in a safe and engaging setting without the inconvenience and stress of travelling. Video conferencing, the web, and chat are commonly used to facilitate synchronous online learning. *Asynchronous learning* allows students to access the learning environment at any time; students may download papers, study at their own pace, and connect with resources, peers, and instructors on their own time. Media such as email and discussion boards are commonly used to assist asynchronous learning [6]. Online learning takes place in a very different environment than traditional face-to-face learning. Learners and teachers in online educational settings are separated by time, location, or both, and communication occurs through video conferencing technologies, forums, chat tools, or email [6]. Therefore, it seems reasonable to pay special attention to investigating student satisfaction in the online learning context.

Deci and Ryan ‘s (2000) self-determination theory (SDT) [7] provides a helpful framework for evaluating students’ preferences and perceptions of online learning [6, 8]. According to the SDT, three basic psychological demands (autonomy, competence, and relatedness) must be satisfied for learners to be motivated and engage in learning environments: Students need to feel self-determination or autonomy in their decisions and a sense of control in the experience. Additionally, they need to believe they are competent and can meet the demands of a particular task. Finally, they must feel socially connected to or included in a group of people. Learners are more likely to be organically motivated if a learning context meets these basic psychological demands, such as by actively engaging in learning tasks, demonstrating improved performance, and demonstrating higher endurance when faced with obstacles [9].

Previous studies that applied the SDT to online learning showed that students’ self-reported motivation and needs satisfaction are positively associated with the quantity and quality of learning behaviours in online educational settings, such as actively posting messages to an online learning platform [6, 8, 10]. Given this theoretical premise, one of the purposes of this study was to assess how students perceive online learning experiences from three domains: preference, effectiveness, and learning satisfaction. We assumed that students who were more likely to accept online tools as valuable and be satisfied with them were more likely to perceive them as easy to use during the COVID-19 pandemic.

### Background context

Prior to the COVID-19 pandemic, all medical and nursing courses in the study setting, King Saud bin Abdulaziz University for Health Sciences (KSAU-HS), were delivered entirely on campus, with face-to-face classrooms for lectures, clinical training in simulation labs, and clinical placements in hospitals, followed by an on-campus final assessment and examination. In addition, some elements of blended learning had already been adopted prior to the pandemic. Blended learning refers to “learning that happens in an instructional context that is characterized by a deliberate combination of online and classroom-based interventions using a variety of instructional resources and teaching methods to activate and support learning” [11]. For example, the students received traditional on-campus learning in the classroom and used Blackboard, a virtual learning environment (VLE), to access learning materials and submit coursework [12].

Due to the COVID-19 pandemic, when social distancing became a significant part of our daily lives, schools and universities were forced to close their doors and convert all their courses to online classes [1, 5]. In March 2020, the COVID-19 pandemic caused considerable problems in the day-to-day activities of education. Like other universities, KSAU-HS has implemented online contingency plans to continue teaching and provide assessments via a digital interface, allowing students to continue their studies while shifting lectures and assessments online. Online lectures, training, and assessments continued through the blackboard system, a powerful online platform, and other programs, such as the Microsoft Teams [12].

### Online learning and headache

Smartphones, computers, and tablets are examples of electronic media with screens as the interface [13]. Online learning typically involves screens, which may not be the best option for people who suffer from headaches [14]. Headache is one of the most common conditions affecting the nervous system, and many of its subtypes that are associated with daily headache syndromes cause severe impairment [15]. Headache is defined as pain in the head that is felt above the eyes or ears, behind the head (occipital), or in the upper back of the neck [16]. Migraines, tension-type headaches, cluster headaches, and medication-overuse headaches are painful and incapacitating symptoms of a small number of primary headache disorders.

A tension-type headache (TTH) is described as pressure or tightness, often like a band around the head, sometimes spreading into or from the neck. A cluster headache (CH) is characterized by frequently recurring (up to several times a day), brief but extremely severe headaches, usually focused in or around one eye, with tearing and redness of the eye; additionally, the nose runs or is blocked on the affected side, and the eyelid may droop. A medication-overuse headache (MOH) is caused by the chronic and excessive use of medication to treat headaches. Migraine is one of the most common headache disorders, lasting from hours to 2–3 days and is characterized by recurring attacks of moderate or severe intensity, with one-sided, pulsating pain aggravated by routine physical activity, and nausea as a primary feature [15]. Due to the increased use of computers for academic work, university students have reported high screen time exposure and the prevalence of headaches [17]. Nonetheless, there are not enough studies that examine the relationship between online learning and screen exposure time and headache.

### Significance of the study

Currently, the emphasis on student success in higher education has shifted to identifying the factors that can be improved. One unresolved issue is student health, specifically regarding headaches, and their impact on student success [18]. Studying headaches among university students is essential, as this population may be more prone to suffer headaches than the general population because of academic factors such as anxiety, stress, inadequate sleep, and improper dietary habits. Similarly, students in the health profession are exposed to factors that trigger headaches, leading to missed study days and poor academic performance [18, 19].

According to the research, excessive screen time is identified as a possible leading cause of eyestrain and headaches. Evidence has linked excessive screen time to adverse outcomes such as irritability, depression, and poor cognitive and socioemotional development, all of which led to poor educational performance [20]. DiSabella (2020) hypothesized that headaches in school could be exacerbated by online learning and compared the prevalence of headaches before and after the COVID-19 pandemic. The results showed that since online learning started, the prevalence of headaches increased from 18% before the pandemic to 42% after the pandemic [21]. Previous research has found a link between screen time and headaches [13, 18, 19]. Likewise, in Saudi Arabia, researchers reported a high prevalence of headaches among Saudi medical students. Ibrahim et al. (2018) found that the prevalence of headaches among Saudi medical students was 53.78%, with the prevalence of migraine headaches being 7.1% [22]. Altalhi et al. (2020) discovered that computer vision syndrome (CVS) is commonly reported among Saudi health sciences students who use various electronic devices, with 68% experiencing headaches due to the use of these devices [23].

However, online learning and headaches have not been studied for university students during the COVID-19 pandemic. Our research aimed to fill this knowledge gap by determining how Saudi health sciences university students perceive online learning and the challenges they face. It also aimed to investigate reported headaches associated with screen exposure time, particularly amid the COVID-19 pandemic. Understanding the online learning experiences of university students and how these experiences may be related to headaches allows for the identification of strategies to improve their online experiences and satisfaction [3].

#### Aim of the study

This study assessed the online learning experiences and headaches associated with screen exposure time among Saudi health sciences university students.

#### Research questions

1. How do university students perceive their online learning experience/satisfaction?
2. What are the challenges that students have reported about online learning?
3. How do students report headaches associated with screen exposure time?
4. Is there an association between online learning and reported headache among Saudi health sciences students?

## Materials and methods

### Study design and setting

This cross-sectional descriptive study was conducted among health colleges' students at KSAU-HS. The cross-sectional design captured the student population at a single point in time and information was gathered using an electronic self-administered questionnaire.

### Subjects and sampling

This study enrolled a convenience sample of health students from three health colleges (the College of Medicine, College of Applied Medical Sciences, and College of Nursing) at KSAU-HS in Jeddah, Saudi Arabia, in 2021. The sampling frame was adopted based on the data obtained from students and academic affairs departments in the three colleges. Students in the preparatory years (first- and second-year students) were excluded from this study because they were studying their preparatory courses at the College of Sciences and Health Professions (COSHP) and had not started their specialized education (medicine, nursing, applied medical science). The sample size was calculated using the Raosoft program. With a confidence interval (CI) level of 95% and a 50% response distribution margin of error of ±5%, the minimum required sample was 323. We received 353 complete questionnaires.

### Data collection instruments

Data were collected using electronic self-administered questionnaires. The questionnaires included the following three sections:

- **Section 1**. Demographic and general information questionnaire. This questionnaire was developed by the researchers and included 12 questions about the students’ college, academic level, age, sex, GPA, BMI, sports practice, extracurricular activities, consumption of junk food, parents’ headache status, tobacco consumption, and sleep quality. Responses were measured using yes/no and multiple-choice options.
- **Section 2.** The online experience questionnaire was developed by Amir et al. (2020) [1] and adapted in the current study to assess students’ preferences and perceptions of online learning and associated challenges during the COVID-19 pandemic. It consists of three parts:
  Part 1: 14 statements that assess three domains: preference (4 items), effectiveness (4 items), and learning satisfaction (6 items). The questionnaire items’ response options represent four Likert-type scales (1 = strongly disagree to 4 = strongly agree). The higher the score, the more satisfied the learner was with the online learning experience.
  Part 2: Three questions about the most effective learning methods, with six online learning format options.
  Part 3: Open-ended question: Students were asked to write the challenges they experienced with online learning. The responses are represented by frequencies and percentages.
- **Section 3**. The screen time exposure and reporting of headaches questionnaire was developed by Montagni et al. (2016) [13]. It is composed of two parts as follows:
  **Part 1-screen time exposure:** This was determined using self-reported average screen time across five different activities:
  1. Using a computer or tablet
  2. On a computer/tablet, playing video games
  3. Using a computer or tablet to browse the internet
  4. On a computer/tablet, watching TV or videos (movies, series, or TV shows)
  5. Making use of a smartphone

Six distinct time categories, ranging from never to more than 8 h, might be chosen. The time spent in front of electronic screens was measured using an unweighted scoring method with an arbitrary six-point scale (0 = never, 1 = less than 30 min, 2 = 30 min to 2 h, 3 = two to 4 h, 4 = four to 8 h, 5 = more than 8 h). “Very low,” “low,” “high,” and “very high” were the four quartiles of the final score [13].

**Part 2-headache baseline assessment:** In this part, participants were asked, “Have you had headache attacks lasting more than 12 hours in the last 12 months?”. Participants who said they had no headaches were placed in the “no headache” category. Participants who said they had headaches were asked to offer more details regarding their symptoms, such as unilateral location, pulsing pain, inhibition of regular activities, exacerbation by routine physical activity, nausea or vomiting, and sensitivity to light or sound. Participants with at least two of the four first symptoms listed above and at least one of the two last symptoms were classified as having migraine; the remaining participants were classified as having non-migraine headaches. Migraine’s participants were also asked if they had any visual, sensory, or motor disturbances before the migraine attack to determine whether they had migraines with or without aura (an aura is a warning sign of a migraine). Finally, the results yielded four distinct groupings (no headache, non-migraine headache, migraine with aura, and migraine without aura) [13].

### Validity and reliability

The study instruments reported high internal reliability in the current study, with Cronbach’s alpha correlation coefficients of 0.80 and 0.888 for the online experience and headache baseline assessment questionnaires. Also, academic experts assessed the content validity of the instruments in their English form. Furthermore, the pre-testing of the tools on 5% of students resulted in no changes to the final instruments.

### Data collection

After receiving the Institutional Review Board (IRB) approval from King Abdullah International Medical Research Center (KAIMRC), the researchers distributed the electronic questionnaire link to participants through their different electronic and social media (sending through email, posting in the blackboard, sending the link through Microsoft Team and WhatsApp groups). The researchers clarified the study’s purpose with complete instructions to all participants and informed consent in the introduction part. The data were collected over two months during the spring semester of the academic year 2020–2021.

### Ethical considerations

The KAIMRC IRB approved the study (SP21J/129/03). The researchers clarified the purpose of the study and the participant’s right to refuse or withdraw at any time without affecting their classes or grades. The researchers obtained informed consent from participants to take part in the study and ensured data privacy and confidentiality.

### Data management and analysis plan

The researchers coded the data and statistically analyzed it using the Social Sciences Statistical Package (SPSS) 25. Demographic characteristics were described using frequency and percentages. Descriptive statistics such as mean and standard deviation were used to summarize the data. Chi-square and Analysis of Variance (ANOVA) were used to compare numerical data. *P*-values of ≤0.05 were considered significant.

## Results

### Demographic information

A total of 353 students participated in this study. The majority were females (67.42%) and aged between 20 and 23 years old (78.75%). Approximately half of them (47.03%) are medical students, (31.44%) are nursing students, and (21.53%) are studying at the college of applied medical sciences. The mean GPA score for the study participants was 4.36 ± 0.5. Of the total sample, 56.37% had average weight, 53.82% do not practice sports nor are involved in extracurricular activities (58.92%). Above one-third of them (40.79%) consume junk food daily, 13.31% are smokers. In terms of sleep quality, half of the students had a good (51.84%) sleep quality while 13.31% had a bad sleep quality. The highest percentage (67.99%) reported that their parent had no history of headache. See supplementary Table 1 for more result.

### Students’ perception of the online learning experience

**Fig. 1:**
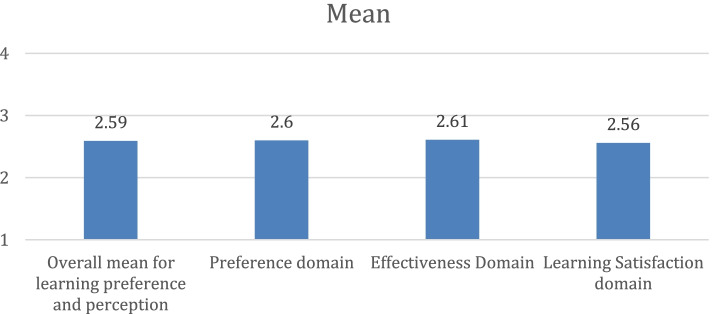
Mean score of Students’ Perception of Online Learning

Figure 1 illustrates the students’ preferences and perceptions of online learning in three domains. The overall mean score of the students’ perception of online learning experience was average 2.59 ± 0.94. Preference domain was presented by a mean score of 2.60 ± 091, while the mean scores were 2.61 ± 091 and 2.56 ± 1.00 for the effectiveness and learning satisfaction domains, respectively.

In the preference domain, slightly above half of the participants (55.8%) preferred that group discussion is more suitably delivered in classroom learning, while assessments (51.56%) and clarification/debriefing sessions (50.0%) are more suitably delivered with online learning. The potentiality of cheating with online learning is a shared concern among high percentage of students (69.98%). In terms of effectiveness, the majority of participants (77.3%) reported that online learning gave more time to prepare learning materials before group discussions, and 71.1% had more time to review all the learning materials after class. On the other hand, 68.56 and 59.77% reported stress and problems during online learning, respectively. For the learning satisfaction domain, the highest percentages (59.3%) agreed that online learning motivates self-directed learning, 53.8% study more efficiently with online learning, and 45.4% prefer online learning to classroom learning. On the other hand, 57.8% of respondents disagreed that online learning is like classroom learning because it facilitates communication with lecturers and fellow students (57.51%). Blended Learning was chosen by more than two-thirds (72.8%). The detailed percentage per item can be found in supplementary Table 2.

#### Effective methods for online learning

Most participants (89.32%) preferred synchronous learning sessions. The highest percentage of students (47.31%) choose synchronous audio-videotext based as the most effective methods for interactive lectures (51.45%), group discussions (45.82%), and group clarification sessions (44.67%) during online learning followed by audio-text based sessions (36.63%). In comparison, text-based synchronous learning was rated as the lowest method (5.38%). The same pattern of results was seen in asynchronous methods of online learning. (Table 1).

**Table 1:** Participants’ perception of the most effective methods for online learning (*N* = 353)

Items	Synchronous			Asynchronous		
	Audio-Video-Text- based	Audio-Text based	Text-based	Audio-Video-Text- based	Audio-Text- based	Text-based
	No. (%)	No. (%)	No. (%)	No. (%)	No. (%)	No. (%)
The most effective method for group discussion	159(45.82)	137(39.48)	12(3.46)	27 (7.78)	9 (2.59)	3 (0.86)
The most effective method for group clarification session	155(44.67)	129(37.18)	21 (6.05)	24 (6.92)	14 (4.03)	4 (1.15)
The most effective method for group interactive lectures	178 (51.45)	115(33.24)	23 (6.65)	19 (5.49)	8 (2.31)	3 (0.87)
Average	47.31%	36.63%	5.38%	6.73%	2.98%	0.96%
	89.32%			10.68%		

### Challenges experienced during online learning

Participants were asked about challenges they experienced with online learning. A total of 247 participants (76.23%) responded to this question. The most frequent challenges they faced were internet connectivity (76.23%), followed by lack of motivation (67.10%), difficulty to focus due to distractions from surroundings (64.61%), and difficulty understanding the content of the subjects (33.69%). See supplementary Table 3 for the rest of the challenges.

### Screen time exposure among participants

Based on the arbitrary six-point scale, the mean time score spent by participants in front of electronic screens was 13.5 ± 3.7 h. The final scores were categorized into quartiles as follow; very low (2.83%), low (35.98%), high (52.69%), and very high (8.50%), indicating that approximately half of the participants (52.69%) had high screen exposure time, and about one third (35.98%) had low screen exposure time. Most of the exposure time was related to working on a computer/tablet (3.7 ± 1.2) and using a smartphone (3.6 ± 1.2). Participants spent from four to more than 8 h in these activities. While they spent less exposure time mean in playing video games on a computer/tablet (1.2 ± 1.3). (Table 2).

**Table 2:** Percentages and mean of the time spent on a screen across five different activities (*N* = 353)

Activities	Never	< 30 min	30 min-to 2 h	2–4 h	4–8 h	> 8 h	Mean ± SD
Working on a computer/tablet	2.83%	2.83%	9.07%	18.41%	37.11%	29.75%	3.7 ± 1.2
Playing video games on a computer/tablet	39.94%	26.06%	17.28%	11.90%	3.12%	1.7%	1.2 ± 1.3
Surfing the Internet on a computer/tablet	7.93%	15.58%	20.96%	25.50%	15.30%	14.73%	2.7 ± 1.5
Watching TV or videos (movies, serials, TV programs) on a computer/tablet	9.63%	12.46%	33.14%	24.93%	13.88%	5.95%	2.4 ± 1.3
Using a smartphone	1.42%	3.12%	12.75%	26.35%	30.03%	26.35%	3.6 ± 1.2
The overall mean time score spent by students in front of electronic screens							13.5 ± 3.7
Total screen time exposure quartiles	**Very low**	**low**		**high**		**Very high**	
	10 (2.83%)	127 (35.98%)		186 (52.69%)		30 (8.50%)	

### Headache baseline assessments among the studied participants

Approximately two-thirds of the participants (65.72%) reported having several hour-long headache attacks in the previous 12 months, compared to the 34.28% who reported no headache attacks. For the first four symptoms, 41.36% of the participants reported pulsating pain, followed by unilateral location (27.76%), the inhibition of daily activities (23.23%), and headache aggravation by routine physical activities (21.81%). Those who reported sensitivity to light or sound accounted for 40.51% of the participants, and 16.71% had nausea or vomiting. Based on these symptoms, 22.09% of the participants were classified as having migraine headaches, as they reported at least two of the first four symptoms and at least one of the last two symptoms, while the remaining participants (44.76%) were classified as having “nonmigraine headaches.” Among the participants with migraines, 41 (11.61%) had migraines with auras, primarily with visual disturbances (82.93%), while 37 (10.15%) had migraines without auras (See Table 3).

**Table 3:** Headache baseline assessment among studied participants (*N* = 353)

Variables	No	%
**Having headache attacks for several hours**		
Yes	232	65.72
No	121	34.28
**The headache symptoms frequently experience**^a^		
• Unilateral location	98	27.76
• Pulsating quality of pain	146	41.36
• Inhibition of daily activities	82	23.23
• Headache aggravation by routine physical activities	77	21.81
• Nausea or vomiting	59	16.71
Sensitivity to light or sound	143	40.51
**Headache status (*N*** **= 232)**		
Non-migraine headache	154	43.63
Migraine:	78	22.09
-Migraine without aura	37	10.15
-Migraine with aura	41	11.61
**Experience any of the following symptoms before the migraine attack (migraine with aura,** ***n*** **= 41)**		
-Visual disturbances	34	82.93
- Sensory disturbances	5	12.20
- Motor disturbances	2	4.87

^a^ *Multiple responses by the same respondent*

### The relationship between the online learning experience and reported headache with screen exposure time

Table 4 revealed a significant relationship between screen exposure time and participant-reported headaches (*p* = 0.046). Nonmigraine headache participants had a high screen exposure time (52.60%). Additionally, migraine participants with aura (63.41%) or without aura (64.86%) had high screen time exposure. In addition, Table 4 shows a significant relationship between the online learning experience and reported headaches (*p* = 0.0003). Students without headaches had a higher overall perception mean of online learning (37.59 ± 8.04). On the other hand, there was no significant relationship between screen exposure time and the online learning experience (*p* = 0.0996).

**Table 4:** The relationship between the online learning experience and reported headaches with screen exposure time

Variables	Frequency and percentages of Screen time exposure				*P*-value
	Very Low	Low	High	Very High	
Headache Category:					0.046^*^
No headache	2 (1.65)	50 (41.32)	55 (45.45)	14 (11.57)	
Non-migraine headache	3 (3.90)	58 (37.66)	81 (52.60)	9 (5.84)	
•Migraine without aura	0 (0.00)	12 (32.43)	24 (64.86)	1 (2.70)	
Migraine with aura	4 (4.88)	7 (17.07)	26 (63.41)	6 (14.63)	
Online learning perception (*Mean ± SD)*	36.60 ± 5.54	37.54 ± 8.11	35.31 ± 7.75	36.33 ± 6.71	0.0996
Preference domain	10.70 ± 2.31	10.63 ± 1.99	10.28 ± 1.75	10.26 ± 1.93	0.3946
Effectiveness domain	10.20 ± 2.39	10.81 ± 3.03	10.19 ± 2.77	10.63 ± 2.37	0.2777
Satisfaction domain	10.70 ± 2.31	10.63 ± 1.99	10.28 ± 1.75	10.27 ± 1.93	0.3946
Online learning	**Headache status**				***P*-value**
	No headache	non-migraine headache	Migraine without aura	Migraine with aura	
Online learning perception	**37.59 ± 8.04**	36.73 ± 7.68	34.51 ± 7.06	31.90 ± 6.47	0.0003^**^

* chi-square **ANOVA

## Discussion

This study focused on assessing the online learning experience and the association of headaches with screen time exposure among Saudi health university students during the COVID-19 pandemic. With the answers to the first questions, the present study revealed moderate participants’ perceptions of the overall online learning experience and its three domains (preference, effectiveness, and learning satisfaction) despite the lower percentage of preferences for online learning (45.4%) compared to classroom learning that was observed in this study. It is worth noting that the students agreed that online learning was more flexible and helped them improve their time management skills by allowing them to more efficiently prepare and study materials, complete assignments, record and review lectures, and encourage self-directed learning. Most participants in the current study preferred Synchronous learning sessions, especially audio-video-text-based sessions, as the most effective methods for interactive lectures, group discussions, and group clarification sessions during online learning, followed by audio-text-based sessions. Additionally, they preferred assessments and clarification/debriefing sessions with online learning. The same results were reported by Amir et al. (2020) [1]. Such findings are consistent with those of Sadeghi (2019) who demonstrated that online learning provides greater flexibility in the location of the study process, saving time and money because commuting to and from campuses is no longer required [24]. Such advantages have been beneficial to students’ learning processes in recent decades, as they must digest an increasing number of new and updated topics [25]. In comparison, Chung et al. (2020a) revealed that most students preferred online learning via pre-recorded lectures because they could listen to the lecture before their classes and replay recorded lectures to better understand the content and prepare for quizzes, tests, and final exams [2].

On the other hand, most respondents disagreed that online learning is similar to classroom learning or facilitates communication with lecturers and fellow students. The potential of cheating with online learning is a shared concern among the highest percentages of students, and they experience stress and problems during online learning. Slightly more than half of the participants preferred group discussion in a classroom setting. Similar findings were reported by Amir et al. (2020) [1]. Additionally, participants in the study by Chung et al. (2020b) preferred classroom learning and stated that they would not continue with online learning in the future if given the option [3]. In this context, Giudice, Antonelli, and Bennardo (2020) stated that the situation is still changing during the COVID-19 pandemic. It is crucial to design the most appropriate learning method for the current situation and have an appropriate plan in place once classroom teaching can resume, taking all necessary safety and health protection protocols into account [26].

For the second question regarding challenges that the participants had experienced with online learning, the current study revealed that internet connectivity, a lack of motivation, difficulty focusing due to distractions from their surroundings, and difficulty understanding the content of the subjects were the most frequent challenges the students faced. Peer-to-peer communication was sometimes lacking among the students, and group discussion interactions were not always possible in the virtual learning method. These difficulties may contribute to the stress felt by most online learners and have some disadvantages. Amir et al. (2020) and Chung et al. (2020a) reported similar challenges [1, 2] and found that increased distractions, complicated technology, limited social interaction, and increased difficulty staying in contact with instructors are all factors that may impede the success of students with online learning. In this regard, Chung et al. (2020a) suggested that universities hold more training sessions to better equip lecturers to deliver online learning content and interactive strategies and improve subject matter understanding [2].

Consistent with our theoretical premise of the STD, one of the most important implications of these findings, we emphasized the importance of considering students’ experiences and preferences to improve students’ motivation, engagement, and perceived positive learning outcomes. The self-determination theory (SDT) provides teachers with practical recommendations on the interactions students require in their learning context, such as enabling choice about content or task performance, providing informational feedback, and assigning group assignments [6]. Similarly, Hsu et al. (2019) showed that meeting students’ basic learning needs increases self-regulated motivation and is linked to improved perceived knowledge transfer and higher achievement of learning objectives [10].

Furthermore, the participants preferred to continue blended learning. The nature of the subjects and curricula of medical and health professions, which combine theoretical understanding and clinical applications with online teaching, is a likely explanation for this preference. Most likely, the students found it challenging to learn subjects solely through online means. Concurrent with our findings, Kiviniemi (2014) discovered that students’ evaluations of the blended approach were very positive, and they preferred the blended learning approach because it offers a mix of pedagogical techniques, delivery mechanisms, and student engagement strategies [27]. In a systematic review, Liu et al. (2016) demonstrated that blended learning might positively affect knowledge acquisition across many students and disciplines directly related to health professions. As a result, future research should evaluate the effect of blended learning, particularly in comparison to traditional learning [28].

### Screen time exposure and reported headaches among the participants

In responding to the third question, approximately two-thirds of the participants had several-hour headache attacks, and 11.61% had migraines with auras, mainly visual disturbances, compared to the 10.15% who had migraines without auras. As previously stated, headache is a common health issue among health professional students, and it can negatively affect their academic performance and quality of life. This result could be related to the numerous physical and psychological stressors they experienced because of their COVID-19 situation, online learning, and preparation for multiple theoretical and clinical exams. Supporting this perspective, Karvounides et al. (2021) cited many factors, including poor ergonomics, the stress associated with uncertainty and time management, the disruption of routine and sleep schedule problems, a stressful home environment, food insecurity, and increased isolation due to the COVID-19 pandemic that may have worsened headaches [17]. Similarly, Desouky, Zaid, and Taha (2019) and Gu and Xie (2018) mentioned that most of these factors are associated with headache prevalence [29, 30].

Several studies have found a diverse prevalence of headaches and migraines among health profession students nationally and internationally. In Saudi Arabia, Al-Jabry et al. (2015) discovered that 58% of the students at Taibah University had a history of tension headaches [31]. Almesned et al. (2018) found that headaches were common among medical students at KSAU-HS, with a 53.78% overall prevalence and a 7.1% prevalence of migraines [32]. Alwahbi et al. (2015) reported that the prevalence of migraine headaches among medical students at KSAU-HS was 23.7% [33]. In contrast, Desouky et al. (2019) found that 47.6% of university students had migraine headaches [29]. Additionally, headache affects 73.1% of health professional university students in India, with a 33.3% prevalence of migraines [19]. In contrast, in France, it was reported that 56.3% of students do not have headaches [13].

The disparity in migraine and headache prevalence rates across the countries and samples could be attributed to sociocultural, geographic, genetic, and methodological differences, the sampling criteria, and individual parameters or characteristics [34]. Similar to Gu and Xie (2018), our study recommended that well-designed interventions and practical education for migraine awareness and headache pain relief should be provided to students [30].

### The relationship between the online learning experience and reported headache with screen exposure time

The present study showed that approximately half of the participants (52.69%) had a high screen exposure time. Most of the exposure time was related to working on a computer/tablet and using a smartphone. They spent from four to more than 8 h performing these activities. This result could explain the significant relationship between high screen exposure time and reported headaches among the migraine (with or without aura) participants and nonmigraine headache participants. For the last studied question, a significant relationship was found between the participants’ online learning experiences and reporting no headaches. Students with no headaches had higher overall perceptions of online learning. This result means that satisfaction with the online learning experience is higher among students with a nonheadache status, and the higher the exposure to screens, the higher the reported headache status.

Previous research has suggested two possible scenarios for how screen time may interact with headache and migraine pathophysiology. First, the brightness or frequency of screen band light may directly cause a migraine attack; second, increasing screen time exposure may lower the migraine cascade threshold [35]. In addition, Ranasinghe et al. (2016) stated that the constant shifting and accommodating that the eye and extraocular muscles endure for an extended time causes stress on the muscles and fatigues the eyes, leading to headache [36]. Our findings are consistent with previous research that found a link between screen time exposure and headaches and migraines in people who frequently use digital devices. Increased exposure to screens (including smartphones, tablets, computers, and television) has been linked to an increased risk of migraines in adolescents and young adults [13, 17]. Moreover, it was declared that while the long-term visual effects of online learning are unknown, there has been an increase in the incidence of vision syndromes, such as near-sightedness, with increased computer use and excessive screen time due to the COVID-19 pandemic [14]. Furthermore, across multiple studies, headaches were the most reported CVS symptoms because of the use of computers and electronic devices [22, 36]. It was also discovered that most students do not use ergonomic practices. As a result, more efforts should be directed towards educating students on the proper use of electronic devices [30].

### What does this study add?

This study contributes to the literature by presenting important information on university students’ current online learning experiences and associated aspects in a Saudi context during the COVID-19 pandemic. Our findings indicate a link between students’ experiences of online learning in terms of preference, effectiveness, and satisfaction and other outcome variables, such as headaches and screen exposure duration. These data could be used to improve and develop future online learning tactics and headache and migraine prevention measures associated with online learning and screen exposure duration. We focused on online learning during the 2020 lockdown. Even if the post-COVID-19 classroom will be different from the circumstances encountered during the first lockdown, the experience has produced insights about the opportunities, potentials, and risks of digitally structured classrooms.

Experiences with online education in real-world settings must be integrated with the existing findings from systematic research on online learning to develop future higher education online teaching and learning. Researchers should keep in mind that demographic, environmental, contextual, and cultural variables, in addition to individual needs, may alter students’ learning experiences [8]. Furthermore, we found the SDT to be a good theoretical model for assessing learning experiences and needs, and we would encourage additional studies to add to the empirical evidence of the SDT in higher education, particularly for online learning.

In addition, the following section will highlight specific limitations and implications to be considered in higher education.

### Limitations of the study

This study, however, had some limitations. First, like other studies on online learning experiences owing to the pandemic, the results relied on data gathered from a single Saudi university; thus, the conclusions can only be extended to a limited population. On the other hand, the university offers various fields and study options. Saudi universities are similarly equipped in basic infrastructure, and the challenges of the COVID-19 pandemic caused a similar disruption in regular education for everyone. As a result, we expect the results to be transferable, at least in the Saudi setting.

Another issue is data quality; there are known issues linked with self-report measures, which are prone to memory distortions. Although self-reports can provide high-quality data for researching motivational, cognitive, or emotional learning components, they should be enhanced with additional data sources [37]. We tried to identify the challenges students faced using an open-ended question. Integrating survey responses from teachers and students, allowing the cross-verification of findings from different perspectives, is still desired in data triangulation for future research on online learning. The frequency and real-time usage of learning management systems (LMSs), chats, or videoconferencing, for example, as well as the number of downloads of recorded lectures, could be used as examples. Another benefit of data triangulation could be the improved integration of qualitative and quantitative data, allowing for more robust confirmation of the results with more focus on the lived experiences of students.

Also, the questionnaire used in this study only measured students’ perceptions and self-reported data on headache status and screen exposure time, both of which were susceptible to recall bias. Another limitation was that the study was cross-sectional, demonstrating the relationship between variables without concluding a cause-effect relationship. We lacked information on screen time exposure conditions, such as the online time between the participants and screens, the screen size, and the possibility of contemporary multiscreen viewing which could be investigated in future research.

## Conclusions

In conclusion, online learning allows for self-paced learning, the adaptability for individual learning needs, and collaborative tasks. This should be accompanied by ongoing technical assistance and high-quality online teaching and learning. As a result, instructors must be enabled to take advantage of digital advancements while also having the freedom to make their own decisions, interact with students and consider the students’ needs [38]. Students in the current study were moderately satisfied with the online learning experience and agreed that online learning is more flexible, helped them improve their time management skills, and motivated their self-directed learning. They preferred synchronous learning sessions, especially audio-video-text-based sessions, as the most compelling method for interactive lectures, group discussions, and group clarification sessions. Nevertheless, the students faced many challenges with online learning that affected their communication efficacy, and they preferred that blended learning be continued. In addition, this study found a high prevalence of headaches (65.72%) and a high screen exposure time among the students who were studied (52.69%). Increased screen time exposure was linked to an increase in reported headaches and migraines among the students. Students who did not experience headaches had a higher overall perception and satisfaction of online learning.

### Implications and recommendations

To overcome the various challenges associated with online learning, the design and delivery of online courses that embrace community, curricula, and assessments and actively engage students in the learning process are critical for an online program’s sustainability and growth. Online learning program instructors must create a nurturing and supportive environment that reduces the stress associated with academic difficulties and peer conflicts and ensure that the communication between faculty and students is constant and practical, including emails, course room postings, and online discussions. Professional development can help teachers prepare for blended learning and integrate technology and learning into an educational environment that promotes interactions, meaningful online lectures, and learning.

To mitigate and prevent headaches and migraines, the National Headache Foundation (NHF, 2020) has recommended a list of tips and precautions that may help avoid headaches associated with screen exposure due to online learning. Frequent breaks from screens are advised to allow the eyes to rest and avoid eye strain; ergonomic chairs should be purchased as uncomfortable seating can cause neck and back pain; meal and sleep schedules should be adjusted; and relaxation and biofeedback techniques should be used to help relieve the stress and anxiety that comes with college life. Moreover, the American Optometric Association recommended angling the computer screen so that it is below eye level and placing one’s feet flat on the floor when using a computer [39]. Alexander (2020) suggested another simple way to reduce the effects of computer vision syndrome in online classes: avoid looking at the screen unnecessarily [14].

In addition to the studies that are highlighted in the limitation section, we recommend future research to compare online learning satisfaction among students from different facilities and geographical locations using multidimensional multi-item instruments and how their intentions to continue using online learning are affected. Academic performance is another area worth investigating because of online learning. Further research is needed to determine whether reducing screen time exposure can help decreasing the frequency of headache and migraine attacks. Data triangulation is another future recommendation, as previously stated in the limitations section.

## Supplementary Information

**Additional file 1: Supplementary Table 1**. Demographic and general information of the study subjects (*N*= 353). **Supplementary Table 2**. Participant’s perception of online learning experience per items (*N*= 353). **Supplementary Table 3**. Challenges experienced with online learning reported by participants (*N*= 247).

## Data Availability

All data generated or analyzed during this study are included in this published article [and its supplementary information files].

## Abbreviations

ANOVA: Analysis of Variance
BMI: Body Mass Index
COVID-19: Corona Virus Disease-2019
CI: Confidence Interval
COSHP: College of Sciences and Health Professions
GPA: Grade Point Average
CVS: Computer Vision Syndrome
IRB: Institutional Review Board
KAIMRC: King Abdullah International Medical Research Center
KSAU-HS: King Saud bin Abdulaziz University for Health Sciences
LMS: Learning Management System
NHF: National Headache Foundation
SDT: Self-Determination Theory
SPSS: Statistical Package of Social Sciences
TV: Television
WHO: World Health Organization
VLE: Virtual Learning Environment
