# Supplementary material for: The online learning experience and reported headaches associated with screen exposure time among Saudi health sciences students during the COVID-19 pandemic
Source: BMC Med Educ. 2022 Apr 1;22:226. doi: 10.1186/s12909-022-03235-8 (PMC8972681; doi:10.1186/s12909-022-03235-8)
Supplement: Supplementary file 1 — Additional file 1: Supplementary Table 1. Demographic and general information of the study subjects (N= 353). Supplementary Table 2. Participant’s perception of online learning experience per items (N= 353). Supplementary Table 3. Challenges experienced with online learning reported by participants (N= 247). [file 12909_2022_3235_MOESM1_ESM.docx]

***Supplementary Table (1) Demographic and general information of the study subjects (N= 353)***

| Variables | Frequency | Percentage |
| --- | --- | --- |
| Gender:   - Female - Male | 238  115 | 67.42  32.58 |
| Age (In Years):   - 20-23 - 24-27 | 278  75 | 78.75  21.25 |
| College:   - Nursing - Applied Medical Sciences - Medicine | 111  76  166 | 31.44  21.53  47.03 |
| Year of study: *   - 3^ed^ year - 4^th^ year - 5^th^ year - 6^th^ year - Internship | 79  98  39  70  67 | 22.38  27.76  11.05  19.83  18.98 |
| Grade Point Average (GPA) score: **   - A - B - C - Mean | 169  146  37 | 48.01  41.48  10.51 |
|  | 4.36±0.5 | |
| BMI:   - Underweight - Normal - Overweight - Obese | 36  199  97  21 | 10.20  56.37  27.48  5.95 |
| Do You Practice Sports?   - Yes - No | 163  190 | 46.18  53.82 |
| Are You Involved in Extracurricular Activities?   - Yes - No | 145  208 | 41.08  58.92 |
| Do You Consume Fast/Junk food in a daily basis?   - Yes - No | 144  209 | 40.79  59.21 |
| Do You Currently Consume Tobacco?   - Yes - No | 47  306 | 13.31  86.69 |
| Sleep Quality:   - Good - Quite good - Neither good nor bad - Bad | 85  98  123  47 | 24.08  27.76  34.84  13.31 |
| Do Your Parents Have a History of Headaches? (Tension-type or Migraine)   - Yes, both - No   Yes, one parent | 24  240  89 | 6.80  67.99  25.21 |

**Each year includes 2 levels; e.g: 3^ed^ year includes level 5&6. The 5^th^ and 6^th^ years are only for the medical students.*

*** Grade A range from 5 to 4.51, Grade B range from 4.5 to 3.51, Grade C range from 3.5 to 2.51*

***Supplementary Table (2) Participant’s perception of online learning experience per items (N= 353)***

| Domains | Strongly agree + agree | Strongly disagree + disagree | Mean ±sd |
| --- | --- | --- | --- |
|  | No. (%) | No. (%) |  |
| Preference domain | | | |
| 1. Assessment is more suitably delivered in online learning | 182 (51.56) | 171 (48.44) | 2.60±0.91 |
| 1. Online learning gives the opportunity for students to cheat during assessment | 247 (69.98) | 106 (30.03) |  |
| 1. Group discussion is more suitable delivered in classroom learning | 197 (55.8) | 156 (44.2) |  |
| 1. Clarification/debriefing sessions are more suitably delivered in online learning | 178 (50.43) | 175 (49.58) |  |
| Effectiveness domain | | | |
| 1. I do not experience stress during online learning | 142 (40.23) | 211 (59.77) | 2.61±0.91 |
| 1. I do not experience any problems during online learning | 111 (31.45) | 242 (68.56) |  |
| 1. I have more time to prepare learning materials before group discussions with online learning | 273 (77.34) | 80 (22.66) |  |
| 1. I have more time to review all of the learning materials after class with online learning | 251 (71.1) | 102 (28.90) |  |
| Learning satisfaction domain | | | |
| 1. Online learning gives similar learning satisfaction to classroom learning | 149 (42.21) | 204 (57.79) | 2.56±1.00 |
| 1. Blended learning (combination of classroom and online learning) can be implemented | 257 (72.80) | 96 (27.2) |  |
| 1. Online learning give motivation for self-directed learning and eager to prepare learning materials before group discussion | 209 (59.21) | 144 (41.8) |  |
| 1. Communication with lecturers and fellow students is easier with online learning | 150 (42.49) | 203 (57.51) |  |
| 1. I like online learning more than classroom learning | 162 (45.89) | 191 (54.11) |  |
| 1. I study more efficiently with online learning | 190 (53.83) | 163 (46.18) |  |
| Overall mean for learning preference and perception | | | 2.59±0.94 |

*Responses of Strongly agree and agree responses were combined; strongly disagree and disagree were combined*

***Supplementary Table (3) Challenges experienced with online learning reported by participants (N=*** 247***)***

| Challenges experienced with online learning* | Frequency | Percentage |
| --- | --- | --- |
| Internet connectivity | 247 | 76.23 |
| Lack of motivation due to absence of face-to-face contact with friends and lecturers | 206 | 67.10 |
| Difficult to focus due to distractions from my surroundings | 199 | 64.61 |
| Difficult to understand the content of the subjects | 94 | 33.69 |
| Too many different online learning methods used by different lecturers | 71 | 26.49 |
| Slow personal laptop, devices | 66 | 26.40 |
| Lack of technical skills in using online learning | 56 | 21.46 |

* *Multiple responses by the same respondent*
